# Development of a Millet Starch Edible Film Containing Clove Essential Oil

**DOI:** 10.3390/foods9020184

**Published:** 2020-02-13

**Authors:** Alaa G. Al-Hashimi, Altemimi B. Ammar, Lakshmanan G., Francesco Cacciola, Naoufal Lakhssassi

**Affiliations:** 1 Food Science Department, College of Agriculture, University of Basrah, 61004 Basrah, Iraq; dr.alaagh@yahoo.co.uk; 2 Central Research Laboratory, Meenakshi Academy of Higher Education and Research, 600078 Chennai, India; lakshmanang261988@gmail.com; 3 Department of Biomedical and Dental Sciences and Morphofunctional Imaging, University of Messina, 98125 Messina, Italy; cacciolaf@unime.it; 4 School of Agricultural Sciences, Southern Illinois University at Carbondale, Carbondale, IL 62901, USA; naoufal.lakhssassi@siu.edu

**Keywords:** millet starch, edible film, Clove, GC-MS

## Abstract

Medicinal plants contain various secondary metabolites. The present study analyzed the essential oil of buds from clove (Syzygium aromaticum L.; Family: Myrtaceae) using gas chromatography-mass spectrometry (GC-MS). GC-MS analysis showed the presence of six major phytoconstituents, such as eugenol (66.01%), caryophyllene (19.88%), caryophyllene oxide (5.80%), phenol, 2-methoxy-4-(2-propenyl)-acetate (4.55%), and humulene (3.75%). The effect of clove essential oils (CEO) at 0%, 1%, 2%, and 3% (*w*/*w*) on the mechanical and barrier properties of starch films was evaluated. The tensile strength (TS) and elongation (E) of films with clove essential oil were 6.25 ± 0.03 MPa and 5.67% ± 0.08%, respectively. The antioxidant activity of the films significantly increased the millet starch film and presented the lowest antioxidant activity (0.3%) at a 30 minute incubation for the control sample, while increasing CEO fraction in the starch film lead to an increase in antioxidant activity, and the 3% CEO combined film presented the highest antioxidant activity (15.96%) at 90 min incubation. This finding could be explained by the incorporation of clove oil containing antioxidant properties that significantly increased with the incorporation of CEO (*p* < 0.05). A zone of inhibition ranging from 16 to 27 mm in diameter was obtained when using a concentration of CEO ranging from 1% to 3%. We also observed the presence of an antimicrobial activity on several tested microorganism including *Escherichia coli*, *Pseudomonas aeruginosa*, *Enterobacter sp, Bacillus cereus*, *Staphylococcus aureus*, and *Trichoderma fungi*. Thus, the current study reveals the possibility of using a millet starch edible film as a preservation method.

## 1. Introduction

In recent years, several investigations focused on the development of eco-friendly, edible, and bio-degradable films using natural resources. Materials that are recognized as safe substances were used, such as lipids, proteins, and polysaccharides, in order to develop edible film and coatings [1]. These materials can be consumed and work effectively as a barrier layer between the food and the surrounding environment [2].

Worldwide, medicinal plants are used for folk medicine and also are consumed either directly or indirectly by humans for health benefits. The secondary metabolites are classified into four main groups, including the flavonoids, terpenoids, nitrogen-containing alkaloids, and sulfur-containing compounds. The plant derivatives have been reported to be effective in the treatment of multi-drug resistance cancer [3,4].

Edible films have several applications, including coloring agents, antimicrobial, and bioactive properties [5]. Moreover, encapsulating antimicrobial substances with edible films can improve the quality of food products and delay the growth of microorganisms [6]. Starch is mostly found in carbohydrates, which play an essential role in people’s diets and exist in granular structures of different sizes and shapes in plants. Starches are composed of two glucan molecules, such as amylose and amylopectin. Starch is also known as biopolymer and it is used as edible film since it could be obtained from wide range of raw materials, in addition to the ability to form films and being produced cheaply. The main reason for developing films of starch is to prevent the changes of taste, color, flavor, and appearance in food products [7].

Approximately 33.692 million hectares of millet was cultivated worldwide, and its production reached 26,702.000 metric tons [8]. Other studies reported that about 33.5 million hectares was used to cultivate millet and about 35 million tons was produced across the world [9]. People with low income could use the millet as an alternative source of carbohydrates because it contains higher amounts of starch [10]. Starch is mainly present in pearl millet and represents 59% to 80% of the endosperm. Barnyard millet contains 66% starch, 15% protein, and 7% lipids, in addition to various micronutrients [11,12]. Starch containing high amylose content is considered as a raw material for edible films presenting good oxygen barrier properties [13]. Some studies reported that starch-based edible films possess the ability to transfer the water vapor, and for this reason, waxes, lipid additives, and essential oils were used in order to improve the hydrophobic fraction side of the film [14].

Essential oils play an important role in antimicrobial activities due to their valuable composition from phenols and terpenes [15]. The synergistic effects between the essential oils and their components can enhance the functional properties of edible films and thereby increase the shelf-life of food production, especially the food containing high fat levels. Earlier studies used essential oils as a potential source to preserve food from deterioration. For instance, using essential oils can cause many problems related to toxicity, intense aroma, and change in the sensory properties of food products [14].

Gas chromatography (GC) analysis is used to analyze biological material containing volatile constituents [16]. Mass spectrometry is a powerful analytical technique for the identification and quantification of known and unknown compounds in order to reveal the structure and chemical properties of molecules.

The biological activity of clove essential oil (CEO) was studied and was found to have the ability to work as an antioxidant, insecticidal, antifungal, and antibacterial agent [15]. In particular, it has been reported that CEO contains enormous amounts of bioactive compounds, such as triterpenoids, sesquiterpenes, and tannins. In addition, some studies reported that one of the main components, *viz.* eugenol (4-allyl-2-methoxyphenol), works effectively as antifungal activity agent [17,18]. Eugenol is used as a food additive and classified to be a safe substance according to the United States Food and Drug Administration (FDA) [19].

The aim of the present study was to study the effect of millet starch-based films and characterize the physical properties as well as antioxidant activity and antimicrobial of the millet starch (MS) films when incorporated with CEO.

## 2. Material and Methods

### 2.1. Materials

Millet, in addition to the source of starch and the clove (*Syzygium aromaticum*) variety used in this study, were purchased from the Basrah local market.

### 2.2. Starch Extraction

Millet starch was extracted according to the method of Bhupender et al. [20]. Millet grains were soaked in distilled water/sodium azide (0.01%) at ratio (1:2) for 24 h at 4 °C to reduce microbial growth. The excess water was drainage steeped and the washed grains were ground in a warming blender with sufficient water. The slurry was sieved on an 85-mesh nylon bolting cloth. The remaining parts (millet peels, germ, and endosperm) were again slurred with water to float off the germ and peels. The grinding, sieving, and regrinding processes of the remnant’s endosperm particles were repeated until they were basically free from starch. The starch–protein slurry was centrifuged at 2000 rpm for 20 min. The supernatant was discarded, and the protein layer on the top of the starch was removed with a spatula. The starch was washed repeatedly by re-dispersing in distilled water and centrifuging until it appeared clean. The cleaned starch was air-dried on a glass plate for 12 h, re-dispersed in water, and wet-sieved through a 100-mesh screen. The starch passing through the screen was recovered by centrifugation (LMC, 3000) (2000 rpm, 20 min) and dried in a hot air oven at 40 °C.

### 2.3. Extraction of Essential Oil

The fresh buds of cloves were washed, and the wet samples were dried in a 30 °C ventilated drying oven. A total of 2 kg of cloves sample were mixed with 5 L distilled water for hydro-distillation by Clevenger apparatus (LG-6656-100, Wilmad, Ottaw, ON, Canada). The mixture was heated in a vertical hydro-distillation unit at 100 °C for 24 h. The CEO was separated from condensed vapor through an auto oil/water separator [21].

### 2.4. Gas Chromatography-Mass Spectroscopy (GC-MS) Analysis

A GC-MS analysis of essential clove oil was carried out using the GC-MS electron impact ionization (EI) method on gas chromatography (Shimadzu) coupled with a GC-MS QP2010 plus Mass Spectrometer (Tokyo, Japan) with an auto-sampler (AOC-20S) and an auto-injector (AOC-20i). A fused silica capillary column HP5-MS (30 m × 0.32 mm, film thickness 0.25μm) was used. One microliter of sample was injected into the capillary column. Helium was the carrier gas at constant pressure of 100Kpa. The flow rate was 1 ml/s and the injector’s temperature was 250 °C. The column temperature starts at 50 °C, settles for 3 min, and then increases by 15 °C every minute until it reaches 250 °C and remains at this temperature for 5 min. The components of the CEO were identified by comparing the spectra with those of known compounds stored in the National Institute of Standards and Technology (NIST) library (2005).

### 2.5. Preparation of the Starch Edible Film

The edible films were prepared according to Resianingrum [22] with a few modifications. Briefly, 5 grams of millet starch were dissolved in 150 mL of distilled water. The starches were melted using a continuous heated stirrer at 75 °C until the solution gelatinized to allow leaching. A total of 2 mL of glycerol was used as plasticizer and then mixed and heated at 60 °C for 30 min. After the heating process was completed, the mixture was cooled down to 30 °C. Next, CEO at three different concentrations (1%, 2%, 3%) was added slowly to the solution with continuous stirring. Approximately 40 mL of the film starch solution was poured onto glass plates, and then fixed to remove the outer rim in all four outlines. The glass plates were covered with aluminum foil. These plates are left until the solution was tightened for 5 h of drying at 75 °C.

### 2.6. Film Thickness

The film thickness was measured using a micrometer to the nearest 0.01 mm of accuracy at five random positions of the films.

### 2.7. Mechanical Properties

Mechanical properties, tensile strength, and elongation were measured using a Universal Testing Machine COM-TEN testing machine 95T series model at the polymer research center/Basrah University. The tests were carried out according to the American Society for Testing and Materials (ASTM) [23].

### 2.8. Water Vapor Permeability

The permeability of films for water vapor was determined according to ASTM [24] with some modifications. The cylindrical cup slot was coated by the circular film samples and was well established using rubber bands. These sylider contained 50 g CaCl_2_ (0% relative humidity, RH) to preserve an RH difference of 75% through the film. The cell was kept in a desiccator at 25 °C containing a saturated NaCl solution (75% RH). The weight of each penetration vessel was recorded after 24 h and the water vapor permeability (WVP) of the films was calculated using the following Equation (1):

(1) $$WVP=\;\frac{\Delta W\;\times \;X}{t\;\times \;A\;\times \;\Delta P}$$

where WAP is the water vapor permeability (g.mm/m^2^.day.kPa); ΔW is the weight gain by going down (g).

### 2.9. Oxygen Transmission Rate

The oxygen transmission rate (OTR) of the millet starch films incorporated with CEO was determined at 23 °C and 50% ± 1% RH according to ASTM [24]. The films were placed on an aluminum foil mask with an open area of 5 cm^2^. The transported oxygen through the films was performed by the carrier gas (N_2_/H_2_) and the colometric sensor. Measurements were made on hourly affinity to reach the stable state of oxygen transport. The permeability coefficients in cc-μm/ (m2day atm) were calculated on the basis of the oxygen transmission rate in a steady state, taking into consideration the thickness water solubility of films.

Three discs of the films were cut into pieces with a 2 cm diameter, weighed, and submerged in 50 mL of distilled water; then, they were slowly moved and periodically agitated for 24 h at 25 °C. The dry mass content of the primary and final samples was determined by drying the samples at 105 °C for 24 h [25].

### 2.10. DPPH Radical Scavenging Activity

The radical scavenging activity of the millet starch films enriched with CEO was estimated according to the method described by Maizura et al. [26]. The antioxidant activity of the films was determined using the DPPH (2,2-diphenyl-1-picrylhydrazyl) free radical scavenging assay. Briefly, 3 mL of the film solution was blended with 1mL of 1 mM methanol solution of DPPH. The mixture was homogenized by a magnetic stirrer and incubated in the dark at an ambient temperature for 30 min. The absorbance was measured against Methanol (blank) at 517 nm, and the percentage of DPPH radical scavenging activity was achieved by the following Equation (2):

(2) $$\mathrm{DPPH}\;\mathrm{scavenging}\;\mathrm{effect}\;\%\;\frac{Abs\;\mathrm{DPPH}\;–\;Abs\;Extracty}{Abs\;\mathrm{DPPH}}\;\times 100$$

where *Abs DPPH* is the blank absorbance value at 517 nm of the methanol solution of DPPH. Abs extract is the absorbance value at 517 nm for the films sample.

### 2.11. Antimicrobial Activity Test

Different isolated microorganisms were obtained from Basra University/the College of Agriculture/the Food Science Department and were used in this study, including *Esherichia coli, Staphylococcus aureus, Pseudomonas aeruginosa, Enterobacter sp., Micrococcus roseus, B. cereus*, and the mold *Trichoderma* to detect the antimicrobial activity of edible film incorporated with CEO. The films were tested for their inhibition against the target microorganisms according to Tooraj et al. [27]. The edible films were cut into 6-mm diameter discs and then put on nutrient agar plates, which were previously inoculated with 0.2 mL of inoculums containing approximately 10^5^–10^6^ CFU/ml of the bacteria. Then, the inoculum was spread in a circular motion until all the liquid was absorbed. The plates were then incubated at 37 °C for 24 h. As for *Trichoderma* sp, Malt extract agar petri dishes were prepared and seeded with the mold culture, then incubated at 22–25 °C for 2–5 days. Next, the plates were examined for a zone of inhibition on the film discs.

### 2.12. Statistical Analysis

The statistical analysis was carried out, employing the Statistical Package for the Social Sciences (SPSS) program using analysis of variance (ANOVA) to investigate the effect of CEO. The obtained result indicates a significant difference for the addition of the Cloves’ essential oils and their concentrations.

## 3. Results and Discussion

### 3.1. Chemical Composition of Clove Oil

**Figure 1:**
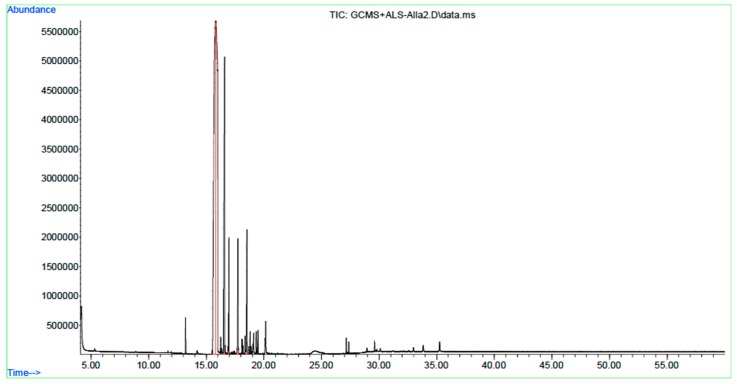
A typical gas chromatogram of the constituents of CEO.

Using the steam distillation method with an average yield of 5.33%, GC-MS analysis (Figure 1) detected the presence of the chemical components and composition of CEO (*Syzygium aromaticum*), which are shown in Table 1. The GC-MS analysis showed the presence of various secondary metabolite, namely eugenol (66.01%), caryophyllene (19.88%), caryophyllene oxide (5.80%), phenol, 2-methoxy-4-(2-propenyl)-acetate (4.55), and humulene (3.75).

The obtained results are in agreement with Chaieb et al. [28] who reported eugenol (88.58%) as the major compound, followed by eugenol acetate (5.62%), and β caryophyllene (1.39%). However, 2-heptanone, ethyl hexanoate, humulenol, α-humulene, calacorene, and calamenene were detected but with very little amount (<1%). Similar results were also reported by Uddin et al. [29], where 3-Allyl-6-methoxyphenol *viz. m*-eugenol (69.44%) was detected as the major constituent, followed by eugenol acetate (10.79%), 4-hydroxy-4-mehtylpentan-2-one viz. Tyranton (7.78%), caryophyllene (6.80%), and 1,4,7-cycloundecatriene, 1,5,9,9-tetramethyl-,Z,Z,Z.

The variation of physical and chemical properties of CEO depends on several factors, including the tissue and origin of the plant, growing season, weather, harvest time, and air humidity. Another important factor is the time between the raw material harvest and oil production. More than 100 ingredients of CEO have been detected worldwide [30].

Eugenol (C_10_H_12_O_2_) (Figure S1) is a volatile phenolic component and is the primary component found in the extracted clove buds’ essential oil [31]. It has a molecular weight of 164.2 g/mol with a peculiar spicy aroma [32]. CEO also presents other components of terpenes, e.g., the ester form of eugenol (Phenol, 2-methoxy-4-(2-propenyl)-acetate) (Figure S2), with documented beneficial properties, including antibacterial [33], antifungal [34], antioxidant [35], and anti-inflammatory [36].

(−)-β-caryophyllene (Figure S3), is a common ubiquitous bicyclic sesquiterpene in nature, and is composed of many essential oils including the extracted oil from the stalk and buds of *Syzygium aromaticum* (cloves) [37].

(−)-β-caryophyllene and its derivative oxide have a severe woody aroma and are utilized as cosmetics in food manufacturing. These two natural components are used as flavors by the Food and Drug Administration (FDA) and by the European Food Safety Authority (EFSA) because of their low toxicity and low water solubility [38]. These compounds were also known to have antimicrobial and antioxidant properties [39], an anti-inflammatory activity against carrageenan and prostaglandin [40], and enhance skin penetration [41].

Humulene (Figure S4), which is known by other designations as α-humulene or α-caryophyllene, is a ring-opened isomer of β-caryophyllene oxide (Figure S5). It occurs in nature as a monocyclic sesquiterpene (C_15_H_24_), including an 11-membered ring, and it is composed of three isoprene units, including three not associated C=C double bonds, two of which are being replaced by a triple bonds [42]. The sesquiterpene hydrocarbon plays a very important role as an antimicrobial activity [43]. It also has a strong anti-inflammatory activity equal to dexamethasone [44].

### 3.2. Physical, Mechanical, and Barrier Properties of Millet Starch Edible Films Incorporated with Cloves’ Essential Oil.

The importance of the mechanical properties of the edible film is due to its beneficial effects during trading and storage; therefore, it is one of the standards that specify the firmness of the film and its ability to promote food safety in food packaging applications, as films need to be strong, durable, and flexible [45].

In the application of polymer film packaging, thickness is an important aspect that requires special attention regarding the material design. The biomass thickness significantly affects other important properties, such as strength, elasticity, and moisture content. The addition of CEOs on a millet starch-based edible film shows a significant effect on film thickness. Table 2 indicates the highest oil concentrations that created an increase in thickness. Thus, the edible film containing 3% CEO has the highest rate of thickness 0.150 mm. This increase is due to the ultimate ingredient component of the edible film composition, as the film will be very thick and has more thickness than the other formulation [46]. According to Nugroho et al. [47], the increase of material concentration using different components for edible files will result in an increase in film thickness, which is coherent with the results obtained in the current study.

As shown in Table 2, the content of the CEO has an effect on the tensile strength of the edible film. The strength length of the standard edible (without CEO) film was significantly different from the films that were enriched with the CEO. The TS of starch edible films incorporated with CEO decreased gradually as the concentration of CEO increased, ranging from 8.60 to 4.40 MPa. The decreasing value of TS was due to the interactions between the starch molecules [48]. It has been reported that essential oils plasticizing capability leads to the reduction in TS [49]. This result was in agreement with the result reported by Maizura et al. [26] who indicated a reduction in the tensile strength of films made from the starch–alginate film, in which different concentrations of lemon oil were added. Further, compared to control films, the lower mechanical resistance of films containing CEO can be explained by the composition of emulsion films. In those structures, the lipid molecules filled the protein matrix and the interactions between lipid and polar molecules occurred, which seemed to be weaker than the polar molecules of the control films.

Elongation is defined as the percentage change in the length of the membrane from its original length. The results show a change in the elongation of edible films when changing the concentration of clove oil, where the elongation was, in the case of the native starch edible film, 9.3%. However, it became 5.67% using CEO within a concentration of 3%. The potential cause of this increase is linked to the fact that the water-resistant surfactant has affected the hydrogen interstitially of the molecules within starch or starch in water [50]. Increasing the elongation at break is considered a positive affect regarding the flexibility of films, especially for those materials used as a package. This phenomenon is due to the presence of oils that play a role as a plasticizer or a lubricant in the hydrocolloid matrix. The obtained results reveal that the starch millet films enriched with CEO were less resistant with less extension than the film without CEO. This could be explained by the fact that lipids are incapable of maintaining a cohesive and uninterrupted matrix [51].

Low tensile and elongation values were the most common results of essential oils incorporation in bio-polymer matrices. The results of tensile strength and elongation were in agreement with Souza et al. [52], who found that the (*TS*) and (*E*) of films with cinnamon essential oil were 1.05 ± 0.16 MPa and 191.27% ± 22.62%, respectively. A loss of macromolecular mobility was obtained due to the increase in the cinnamon essential oil, glycerol, and emulsifier contents that impacted the TS and E of the films. The obtained data show that the control films (without essential oil) presented higher TS (3.96 ± 0.60) MPa but lower E (123.61% ± 19.57%) [53].

Water vapor permeability is one of the most important factors in the quality of food packaging materials. The applicable film must be able to avoid, or at least reduce water transfer between environment and food. As shown in Table 2, the WVP of the edible films increased proportionally with the concentration of CEO from 1% to 3% (9.67, 12.52 g mm^−2^ d^−1^ KPa^−1^) when compared to the control film, which was 6.92× (g mm^−2^ d^−1^ KPa^−1^). The water vapor permeability of edible films was reduced when the hydrophobic component of the edible film was increased. The hydrophobic compounds can play an important role in reducing the surface tension of the solution. The permeability of membranes made of starch regarding water vapor is influenced by several factors, including temperature, the thickness of the membrane, and the content of the additives [54].

The obtained low WVP rate in starch films on CEO may be due to hydrogen and covalent interactions between the starch network and the polyphenolics compounds. These reactions probably minimize the accessibility of hydrogen groups to form a hydrophilic link with water, resulting in a reduction in the affinity of the film [55].

It is desirable to have a limited exposure of food to oxygen because it can lead to oxidation in addition to sensory changes (odor, color, flavor, and texture) and the loss of nutrition [56]. The results of oxygen permeability of the millet starch edible film with and without CEO are shown in the Table 2. The increased content of CEO caused an increase in the oxygen permeability coefficient (PO_2_) from (24.50 to 28.78) g s^−1^ m^−1^ Pa^−1^ × 10^−10^.

The composition of the matrix affects the proliferation of gas molecules through the polymer, resulting in significant variations in gas transmission. The primary CEO modifies the performance of the barrier, which is associated with the compatibility of starch and oil, eventually leading to the permeation of the effective gas molecule through films [57]. The effect of adding fat on the microstructure of the edible films is a critical factor in barrier efficiency. The physical state of essential oils and their distribution in the polymer matrix impacted significantly the microstructure of films. The liquid state of essential oils can improve molecular movement and promotes the transport of gas molecules [58]. In fact, the authors investigated properties of starch-based edible films incorporated with oregano and black cumin essential oil and indicate that water vapor barrier properties decreased proportionally with essential oils addition [58].

The solubility in water of the prepared starch films with/without CEO is presented in Table 2. The solubility value of the control film was 30.40%, while solubility of the films with CEO addition decreased from 28.67% to 27.13% with 1% and 3% CEO, respectively. When EO was added in starch film, solubility in water decreased. The engagement of EO in the structure of starch and interaction between the CEO and the hydroxyl groups of starch can increase the hydrophobic nature of the films. Therefore, the availability of hydroxyl groups and its interaction with water molecules was reduced and led to less solubility. Perhaps the decrease of starch film with CEO solubility is due to the formation of amylose-lipid complexes with a tight helical structure due to the formed links with the hydrophobic hole [59].

### 3.3. Free Radical Scavenging Activity

A DPPH scavenging assay was employed to elucidate the antioxidant activity of the millet starch films with and without CEO (Table 3). As the concentration of essential oil increased, the DPPH scavenging activity of the films was significantly increased in the millet starch film and presented the lowest antioxidant activity (0.3%) at 30 minute incubation for the control sample, while increasing the CEO fraction in the starch film lead to an increase of antioxidant activity and 3% CEO combined film presenting the highest antioxidant activity (15.96%) at 90 min incubation, which may be due to the presence of eugenol shown as the principal component of buds oil from clove. This was in agreement with the reported highest antioxidant activity of films containing additional eugenol microcapsules [60]. In fact, it has been shown that the addition of eugenol microcapsules containing oleic acid promoted the eugenol retention in the starch matrix during film formation and impacted positively the antioxidant activity. When films were developed with encapsulated eugenol powder containing lecithin and oleic acid, low and constant values of peroxide index, conjugated dienes, and trienes were observed, resulting in a high and effective prevention of sunflower oil oxidation even over seven weeks of storage [60].

The antioxidant activities of these biodegradable films are related to the type and concentration of essential oils. This significant antioxidant capacity of CEO could be attributed to a higher content of phenolic components, such as Eugenol, Caryophyllene, Humulene, and Caryophyllene oxide. Shojaee-Aliabadi et al. [61] reported that phenol compounds are accountable for antioxidant activity in EO. Fernandes de Oliveira et al. [62] also indicated that phenolic compounds are receptors for free radicals by breaking the chain oxidation reactions or by metal clawing, which can be an indicator of the antioxidant capacity of the CEO.

A significant amount of antioxidants in cloves belong to the high content of phenolic compounds, such as eugenol and eugenyl acetate, and their ability to donate hydrogen (which is an effective radical scavenger) [63], ISO-eugenol, and caryophyllene [64], but with lower amounts of benzyl alcohol, chavicol, acetyl salicylate, and humulenes [65].

This finding was in agreement with Dashipour et al. [66], who reported that the antioxidant activity of the carboxymethylcellulose (CMC) film without EO was 0.32%, while the antioxidant activity of CMC film with CEO was 71.76%.

The antibacterial activity of *Syzygium aromaticum* showed maximum activity at different concentrations. The starch edible film containing different concentrations of oils of *Syzygium aromaticum* were screened for antimicrobial activity against five standard bacteria species: one Gram-positive bacteria *Staphylococcus aureus,* and four Gram-negative bacterial strains, including *Escherichia coli*, *Pseudomonas aeruginosa, Enterobacter* *sp,* and *B. cereus,* as well as one standard fungal strain *Trichoderma.*

**Figure 2:**
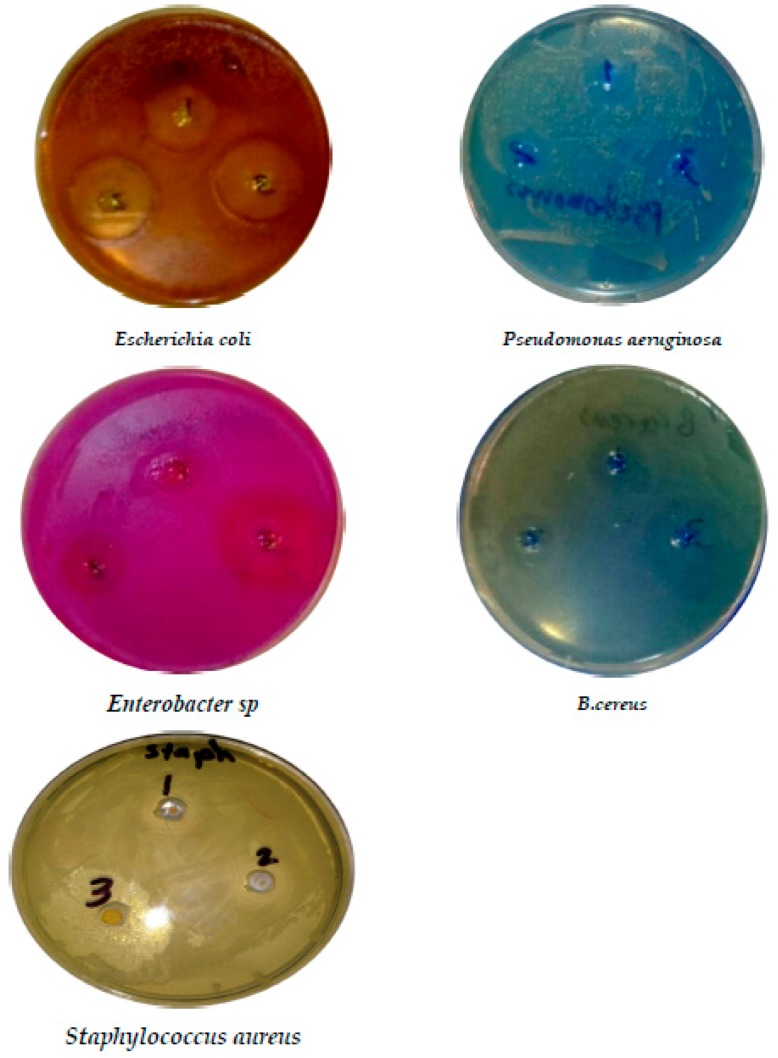
Antimicrobial activity of the MS edible film incorporated with CEO.

The antibacterial activity of the AgNPs was dependent on the concentration. Elevated levels of AgNPs exhibited more inhibitory activity, impacting the growth of microbes. Different results of inhibition were obtained from the millet starch-based film incorporated with different concentrations of cloves’ essential oils and for each microorganism studied (Table 4 and Figure 2). The statistical analysis indicated significant differences among the antimicrobial activity of films with different cloves’ essential oil contents (*p* < 0.05). Certainly, there was no affect against microorganisms for the edible film without CEO (control). The films exhibited strong antimicrobial effects against all tested bacterial strains, including *Escherichia coli, Pseudomonas aeruginosa, Enterobacter sp, B. cereus, Staphylococcus aureus,* and *Trichoderma fungi.* Additionally, 3% of clove oils showed a zone of inhibition ranging from 16–27 mm in diameter. These results show that starch-based edible films incorporated with different CEO amounts have inhibitory efficiency against both positive and negative bacteria, while the inhibitory effect increases proportionally with CEO amount. However, the inhibitory activity of clove oil due to the presence of several constituents was mainly observed in eugenol and eugenyl acetate and β- caryophyllene. These components can change protein structure and the phospholipids of cell membranes by affecting their permeability [67]. The hydrophobic criteria of essential oils interact with the lipid structure, such as Gram-negative bacteria cell membrane, mitochondria, and most intracellular component, which lead to damaging the cell structure, leaching, ion exchange, breathing inhibition, and finally causing cell death [68,69]. These reported phenotypes were coherent with our findings. The positive strain *Staphylococcus aureus* has the lowest sensitivity toward the films’ cloves oil components, especially eugenol, which is the main factor inhibiting fungal activity due of the lyses of spores and micelles [70,71].

## 4. Conclusions

Edible films play an important role in the revelation of packing and keeping the environment safe. Millet starch edible films with CEOs containing polyphenolic components provide new ways to enhance antioxidant and microbial safety and extend the shelf life of foods. The present study showed the presence of significant differences in the mechanical and barrier characteristic since the concentration of clove oil varied from 1–3%. The active volatile and semi-volatile compounds were ascertained by GC-MS analysis. The results also showed that the presence of significant amounts of antioxidants in cloves was due to the high content of phenolic compounds. The current study also revealed that the engagement of EO in the structure of starch and the interaction between the CEO and the hydroxyl groups of starch can increase the hydrophobic nature of the films. The decrease in solubility is most probably due to the availability of hydroxyl groups, which reduces its interaction with water molecules. The study also demonstrated that the major phytoconstituent of the essential oil of clove buds was the eugenol (66.01%). The obtained results are coherent with other studies that showed that encapsulated eugenol modified the film microstructure and yielded less stretchable films with a reduced water affinity, transparency, and oxygen permeability when compared to films formulated with non-encapsulated eugenol [60].

## Figures and Tables

**Table 1:** Percentage composition of volatile constituents of clove essential oils (CEO).

No.	Compound	t_R_ (min)	Molecular Weight	Composition %
1	Eugenol	15.80	164.2	66.01 ± 0.23
2	Caryophyllene	16.59	204.36	19.88 ± 0.11
3	Humulene	16.96	204.35	3.75 ± 0.65
4	Phenol, 2-methoxy-4-(2-propenyl)-acetate	17.74	206.23	4.55 ± 0.17
5	Caryophyllene oxide	18.53	220.35	5.80 ± 0.21

**Table 2:** Tensile strength (TS), thickness (TH), elongation at break (E), water vapor permeability (WVP), oxygen permeability coefficient (PO_2_), and the solubility (S) of millet starch films incorporated with different contents of CEO.

Film	TH (mm)	TS(MPa)	E (%)	WVP	PO_2_	S (%)
Control	0.120 ± 0.003	10.52 ± 0.05	9.3 ± 0.05	6.92 ± 0.088	19.70 ± 0.57	30.40 ± 0.3
MS- films (1% CEO)	0.130 ± 0.008	8.60 ± 0.08	7.43 ± 0.01	9.67 ± 0.088	24.50 ± 0.1	28.67 ± 0.14
MS- films (2% CEO)	0.135 ± 0.001	7.16 ± 0.05	6.25 ± 0.05	11.33 ± 0.033	26.25 ± 0.57	27.50 ± 0.8
MS- films (3% CEO)	0.150 ± 0.008	6.25 ± 0.03	5.67 ± 0.08	12.52 ± 0.08	28.87 ± 0.8	27.13 ± 0.145

**Table 3:** Antioxidant activity of millet starch (MS) edible film incorporated with CEO.

Film	% Inhibition of DPPH ± SD	
	30 Minutes Incubation	90 Minutes Incubation
Control	0.3 ± 0.100	0.7 ± 0.057
MS-films (1% CEO)	13.88 ± 0.075	36.57 ± 0.337
MS-films (2% CEO)	17.50 ± 0.100	57.34 ± 0.020
MS-films (3% CEO)	23.22 ± 0.890	85.96 ± 0.14

**Table 4:** Antimicrobial activity of the MS edible film incorporated with CEO.

No.	Standard Microorganisms	Zone of Inhibition (mm)			
		Concentration (%)			
		Control (0)	1	2	3
Tasted Bacteria					
1	*Escherichia coli*	0	16 ± 0.13	18 ± 0.16	23 ± 0.43
2	*Pseudomonas aeruginosa*	0	12 ± 0.15	14 ± 0.13	24 ± 0.32
3	*Enterobacter sp.*	0	14 ± 0.16	16 ± 0.11	27 ± 0.81
4	*B.cereus*	0	11 ± 0.34	12 ± 0.84	20 ± 0.52
5	*Staphylococcus aureus*	0	10 ± 0.58	11 ± 0.52	18 ± 0.95
Tasted Fungi					
	*Trichoderma*	**0**	13 ± 0.65	27 ± 0.32	14 ± 0.76

## Supplementary Materials

The following are available online at https://www.mdpi.com/2304-8158/9/2/184/s1, Figure S1: MS spectrum of Eugenol; Figure S2: MS spectrum of Eugenol acetate; Figure S3: MS spectrum of caryophyllene; Figure S4: MS spectrum of humulene; Figure S5: MS spectrum of caryophyllene oxide.
