# Supplementary material for: Development of a Millet Starch Edible Film Containing Clove Essential Oil
Source: Foods. 2020 Feb 13;9(2):184. doi: 10.3390/foods9020184 (PMC7074152; doi:10.3390/foods9020184)
Supplement: Supplementary file 1 [file foods-09-00184-s001.pdf]

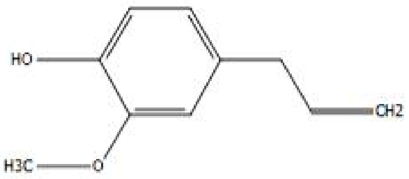

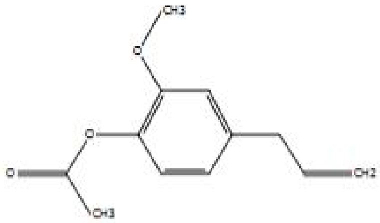

Eugenol acetate

Eugenol

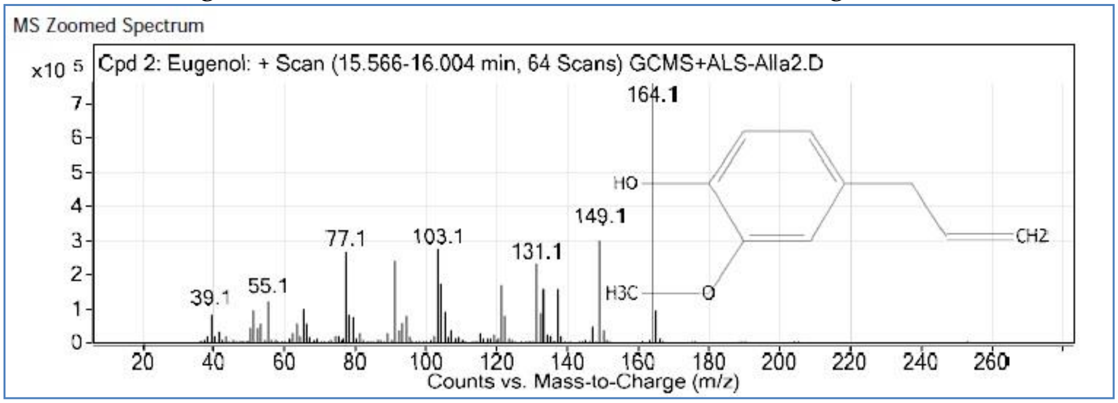

Figure 1. MS spectrum of Eugenol.

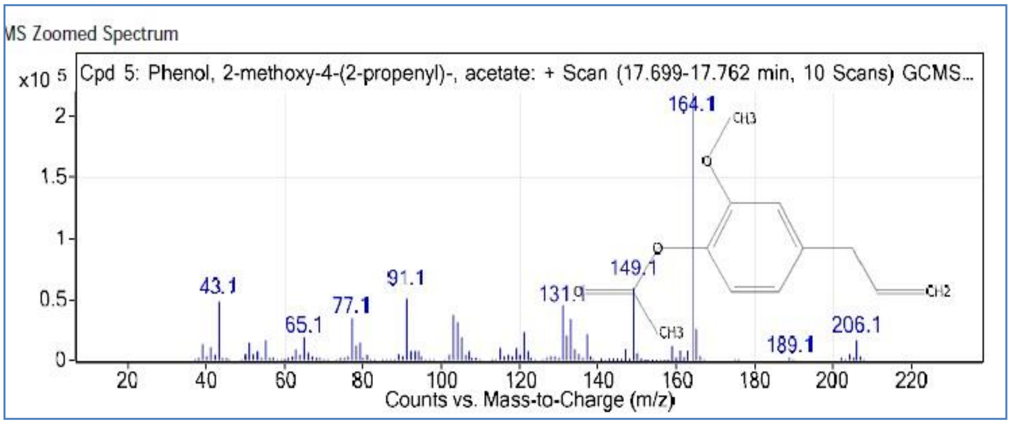

Figure 2. MS spectrum of Eugenol acetate.

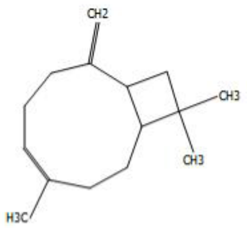

β-caryophyllene

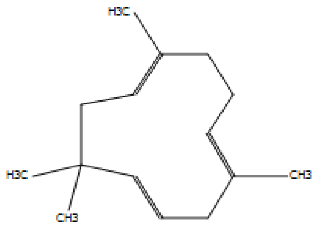

Humulene

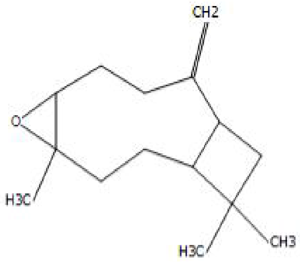

β-caryophyllene oxide

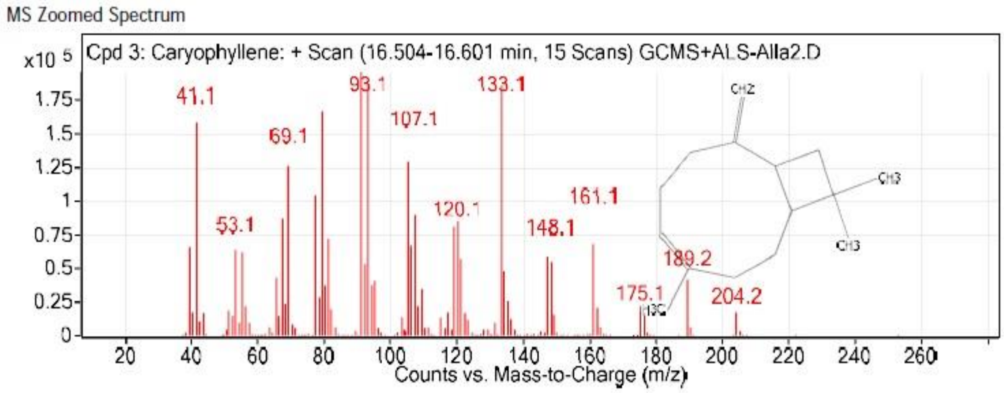

Figure 3. MS spectrum of caryophyllene.

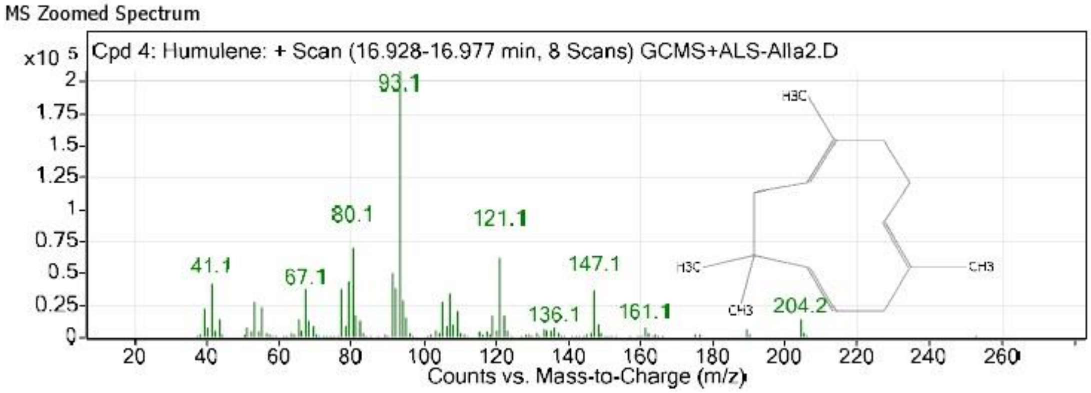

Figure 4. MS spectrum of humulene.

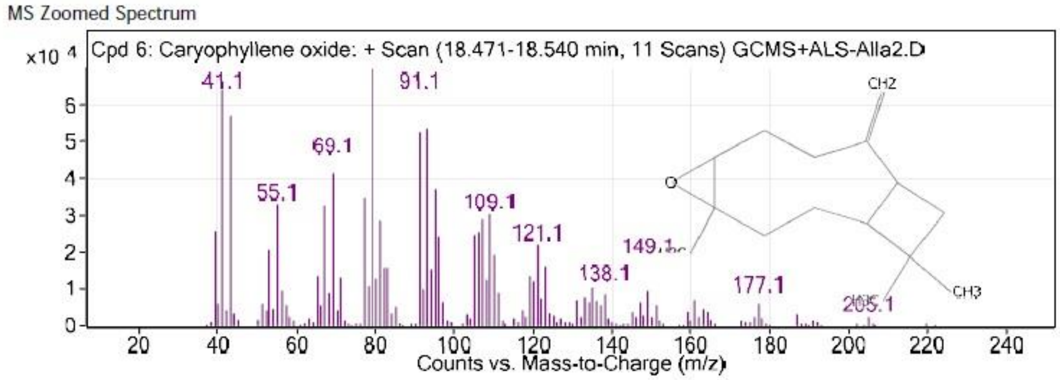

Figure 5. MS spectrum of caryophyllene oxide .
